# Supplementary material for: Empirical methods for controlling false positives and estimating confidence in ChIP-Seq peaks
Source: BMC Bioinformatics. 2008 Dec 5;9:523. doi: 10.1186/1471-2105-9-523 (PMC2628906; doi:10.1186/1471-2105-9-523)
Supplement: Additional File 1 — A variety of html documents from the USeq web site detailing the available applications, their best usage, output file type descriptions, command line menus, etc. [file 1471-2105-9-523-S1.zip › USeqUserGuides/JavaDoc.zip!/JavaDoc/bioroot/strain/NewStrain.html]

NewStrain


|  |  |  |
| --- | --- | --- |
| **Overview** \| **Package** \| **Class** \| **Use** \| **Tree** \| **Deprecated** \| **Index** \| **Help** | |  |
| PREV CLASS   **NEXT CLASS** | **FRAMES**    **NO FRAMES**     **All Classes** |  |
| SUMMARY: NESTED \| FIELD \| CONSTR \| METHOD | DETAIL: FIELD \| CONSTR \| METHOD |  |


---


## bioroot.strain Class NewStrain

```
java.lang.Object
  javax.servlet.GenericServlet
      javax.servlet.http.HttpServlet
          bioroot.strain.NewStrain
```

**All Implemented Interfaces:**: java.io.Serializable, javax.servlet.Servlet, javax.servlet.ServletConfig

---

``` public class NewStrain extends javax.servlet.http.HttpServlet ```

**Author:**
:   nix
    Servlet to process a new strain.

**See Also:**: Serialized Form

---

**Constructor Summary**

`NewStrain()`


| **Method Summary** | |
| --- | --- |
| `static void` | `addParent(StrainBean bean, java.util.HashMap nameValue, DBUtil bioRoot, UserBean userBean)` |
| `static void` | `addPlasmid(StrainBean bean, java.util.HashMap nameValue, DBUtil bioRoot, UserBean userBean)` |
| `void` | `doGet(javax.servlet.http.HttpServletRequest request, javax.servlet.http.HttpServletResponse response)` |
| `void` | `doPost(javax.servlet.http.HttpServletRequest request, javax.servlet.http.HttpServletResponse response)` |
| `static void` | `removeParent(StrainBean bean, java.util.HashMap nameValue, DBUtil bioRoot)` |
| `static void` | `removePlasmid(StrainBean bean, java.util.HashMap nameValue, DBUtil bioRoot)` |

**Methods inherited from class javax.servlet.http.HttpServlet**

`service`

**Methods inherited from class javax.servlet.GenericServlet**

`destroy, getInitParameter, getInitParameterNames, getServletConfig, getServletContext, getServletInfo, getServletName, init, init, log, log`

**Methods inherited from class java.lang.Object**

`equals, getClass, hashCode, notify, notifyAll, toString, wait, wait, wait`

**Constructor Detail**

### NewStrain

```
public NewStrain()
```


**Method Detail**

### doGet

```
public void doGet(javax.servlet.http.HttpServletRequest request,
                  javax.servlet.http.HttpServletResponse response)
           throws javax.servlet.ServletException,
                  java.io.IOException
```

:   **Overrides:**: `doGet` in class `javax.servlet.http.HttpServlet`
:   **Throws:**: `javax.servlet.ServletException`: `java.io.IOException`

---


### doPost

```
public void doPost(javax.servlet.http.HttpServletRequest request,
                   javax.servlet.http.HttpServletResponse response)
            throws javax.servlet.ServletException,
                   java.io.IOException
```

:   **Overrides:**: `doPost` in class `javax.servlet.http.HttpServlet`
:   **Throws:**: `javax.servlet.ServletException`: `java.io.IOException`

---


### removePlasmid

```
public static void removePlasmid(StrainBean bean,
                                 java.util.HashMap nameValue,
                                 DBUtil bioRoot)
```

---


### addPlasmid

```
public static void addPlasmid(StrainBean bean,
                              java.util.HashMap nameValue,
                              DBUtil bioRoot,
                              UserBean userBean)
```

---


### removeParent

```
public static void removeParent(StrainBean bean,
                                java.util.HashMap nameValue,
                                DBUtil bioRoot)
```

---


### addParent

```
public static void addParent(StrainBean bean,
                             java.util.HashMap nameValue,
                             DBUtil bioRoot,
                             UserBean userBean)
```


---


|  |  |  |
| --- | --- | --- |
| **Overview** \| **Package** \| **Class** \| **Use** \| **Tree** \| **Deprecated** \| **Index** \| **Help** | |  |
| PREV CLASS   **NEXT CLASS** | **FRAMES**    **NO FRAMES**     **All Classes** |  |
| SUMMARY: NESTED \| FIELD \| CONSTR \| METHOD | DETAIL: FIELD \| CONSTR \| METHOD |  |


---
